# 

**Published:** 1898-10-01

**Authors:** 


### The Hospital

A JOURNAL OF

THE MEDICAL SCIENCES AND HOSPITAL ADMINISTRATION

INCLUDING

A SPECIAL SECTION FOR NURSES.

EDITED BY

SIR HENRY BURDETT, K.C.B.,

AND

SOLOMON C. SMITH, M.D.

IN TWO VOLUMES ANNUALLY.

**VOLUME XXV.**

October, 1898, to March, 1899.

SURGEON GENL'S OFFICE
16546
LIBRARY.

London:
PUBLISHED BY THE SCIENTIFIC PRESS (LIMITED), AT THE HOSPITAL BUILDING,
28 & 29, SOUTHAMPTON STREET, STRAND, W.C.

MDCCCXCIX.

WI
H069X

### INDEX TO VOL. XXV., 1898–99.

*** Articles under the general heading *Medical Progress and Hospital Clinics* are distinguished by the initial (C.) for *Hospital Clinics*, and by (P.) for the rest of the series. (W.) distinguishes the *Institutional Workshop* articles, and (A.) the *Annotations*.

Abdomen, Contused Wound of the (C.), 97

Abdomen, Penetrating and Non-penetrating Wounds of the (P.), 331

Abdominal Incisions (P.), 331

Abdominal Injuries, Severe, without External Wound (C.), 81

Abdominal Operations, Preparation for, &c. (C.), 209, 227

Abdominal Section as a Cure for Vomiting (P.), 10

Abdominal Surgery (P.), 10, 331

Abdominal Symptoms in Diseases of the Digestive Organs (P.), 49

Abdominal Vessels, Thrombosis of (P.), 66

Abdominal Walls, Grave Injuries to (C.), 329

Acromegaly (P.), 230

Addenbrooke's Hospital, Cambridge, 37, 135, 155, 183

Addison's Disease (P.), 131

Adenoids (P.), 280

After San Juan (W.), 352

Albuminuria in Diabetes (P.), 246

Alcohol, Disinfecting Power of (P.), 382

Alimentary Tract, The (P.), 348, 364

Almshouses, Our National, 357

Anæmia, Treatment of (P.), 229

Anæsthetics, A Grumble about (C.), 311

Analgesia, Local Infiltration (C.), 327

Anastomosis, Intestinal (P.), 67

Aneurism (P.), 146 (C.) 277

Aneurisms, The Extirpation of (C.), 378

Antiseptics (P.), 437

Antiseptics at Guy's Hospital (C.), 64

Antitoxin (Diphtheria) in General Practice (C.), 399

Antitoxin Story, An (A.), 359

Anti-Vivisection Meeting, An (A.), 21

Aorta, Obliteration of the (P.), 380

Aphasia and Testamentary Capacity (P.), 331

Appendicitis (P.), 333

Architectural Art and Sanitation (C.), 432

Army Convalescent Homes (W.), 370

Arterial Blood Pressure, The Measurement of (C.), 328

Arteries, Functional Hypertrophy of the (P.), 382

Arteritis, Tuberculous (P.), 380

Arthritis (C.), 397

Artificial Lighting for Public Institutions (W.), 152, 167

Asthma, Paraldehyde in (C.), 345

Bacteria, Pathogenic, The Saprophytic Life of (A.), 374

Bacteriological Methods of Diagnosis in Private Practice (C.), 7, 80

Bacteriology in France (A.), 429

Bacteriology and the Metropolitan Asylums Board (A.), 44

Belladonna in Broncho-Pneumonia in Children (C.), 313

Bladder (P.), 219

Bladder, Air Inflation of the (P.), 433

Blood, Diseases of the (P.), 130

Book World of Medicine, The (*Books, Magazines, and Pamphlets Reviewed*)

Aarons, Golden Rules of Gynæcology, 334

Austin, Manual of First Aid, 282

Baby Feeding (by a Doctor), 266

Bannatyne. The Thermal Waters of Bath, 420

Brown. The Secret of Good Health and Long Life, 420

Caldwell. *See Nahm*

Catalogue of Patented Sanitary Appliances, 300

Chreiman. Health Loss and Gain, 150

Christian, Princess. *See Esmarch*

Clarkson. Atlas of Histology, 87

Davies. *See Wells*

Dowse. The Treatment of Disease by Physical Methods, 334

Eason's Every-Hour Diary, 216

Edgcumbe-Rogers. Local Government Annual for 1899, 300

Emery. *See Stenhouse*

Eshner *See Jakob*

Esmarch (trans. by Princess Christian). First Aid to the Injured, 70

Fenwick. Golden Rules of Surgical Practice, 334

Ferguson. *See Haultain*

Fleming. The Wanton Mutilation of Animals, 118

Fothergill. Golden Rules of Obstetric Practice, 334

Gadd. Synopsis of the British Pharmacopœia, 1898, 103

Greene. The Schott Treatment of Chronic Heart Disease, 216

Gubb. Handbook of Midwifery, 368

Gudthausen, Von, Sprachführer fur die Arzsliche und Pharmacentitche Praxis, 52

Book World of Medicine (*continued*).

Haig. Diet and Food in Relation to Strength, 166

Harvey. *See Martindale*

Haultain and Ferguson. Handbook of Obstetric Nursing, 150

Homfrey. *See Kingzett*

Humane Science Lectures, 300

Hyde. Buxton: Its Baths and Climate, 12

Jack. *See Pollack*

Jakob. (Ed. by Eshner.) Atlas of Methods of Clinical Investigation, 70

Jones. Handbook to the Workmen's Compensation Act, 1897, 12

Kingzett and Homfrey. Pocket Dictionary of Hygiene, 150

Lane. Herbert Fry's Royal Guide to London Charities for 1899, 334

Lawless. First Aid to the Injured, 52

Lefert. La Pratique des Maladies des Enfants dans les Hôpitaux de Paris, 200

Legge and Sessions. Cattle Tuberculosis, 35

Letts's Medical Diary, 1899, 216

Linn. The Health Resorts of Europe, 87

Luff. Gout, Its Pathology and Treatment, 216

Maddox. Tests and Studies of the Ocular Muscles, 35

Martindale. Harvey and Davidson's Syllabus of Materia Medica, 200

Massey. Conservative Gynæcology and Electro-Therapeutics (P.), 118

Morris. Diseases of the Skin, 234

Nahm. (Trans. by Caldwell.) The Erection of a Consumptive Sanatorium for the People, 300

Petty. Cleanliness in Children, 300

Physician's Book for Private Formulæ, 384

Poland. Skiagraphic Atlas, 118

Pollack. (Trans. by Jack.) Methods of Staining the Nervous System, 134

Purdy. Practical Uranalysis and Urinary Diagnosis, 200

Ravenhill. Elements of Sanitary Law, 87

Rideal. Disinfection and Disinfectants, 200

Schenck. The Determination of Sex, 52

Schofield. The Unconscious Mind, 134

Sessions. *See Legge*

Shaw's Manual of the Vaccination Law, 216

Simpson. Wright's Improved Physician's Visiting List, 134

Sisley. London Water Supply, 368

Sladen. Who's Who, 1899, 234

Slater and Spitta. Atlas of Bacteriology, 87

Spitta. *See Slater*

Spitta. Photo-micrography, 420

Stenhouse and Emery. People's Guide to the Workmen's Compensation Act, 13

Tobin. Synopsis of Surgery, 103

Thompson. The Chemist's Compendium, 35

Tweedie. George Harley, F.R.S., 282

Vulliamy. Fry's Law of Vaccination, 420

Walters. Sanatoria for Consumptives, 384

Weber. Mineral Waters and Health Resorts of Europe, 12

Wells and Davies. Text Book of Zoology, 134

Whitaker's Naval and Military Directory, 420

Whitla. Elements of Pharmacy, 103

Williams. Medical Diseases of Infancy and Childhood, 70

Wilson. Handbook of Hygiene, 166

Winslow. Mad Humanity, 266

Year Book of Treatment for 1899, 384

Boycott, An Organised, 2

Brain, Surgery of the (P.), 101, 115

British Laryngological, Rhinological, and Otological Association (P.), 128

Bromoform (C.), 313

Bullets in the Brain and the Röntgen Rays (P.), 116

Canaries (A.), 207

Cancer of the Breast, Early and Free Operation for (C.), 433

Cancer of the Breast, Oophorectomy for (P.), 215

Cancer of the Breast, Operative Treatment of (C.), 177

Cancer, Mortality from (P.), 199

Cancer, Primary, of the Pancreas (P.), 246

Cancer of the Uterus, Operation in (C.), 161

Carcinoma, Inoperable, Treatment of (P.), 214

Carcinoma, Metastatic, of Choroid (P.), 198

Cardio-Pulmonary Murmur (P.), 381

Care of Semi-Lunatics, The, 255

Catching Trains (A.), 44

"Cassaripe" in Infectious Eye Diseases (P.), 403

Cerebro-Spinal Fluid Escaping from the Nose (C.), 112

Chances of Life in Severe Abdominal States (P.), 333

Chilblains, The Electric Bath for (C.), 277

Children, Diseases of (P.), 9, 50, 114

Children's Christmas Parties, 271

Chloroform Administration in the Midlands (C.), 211

Chlorosis: Its Complications and Treatment (C.), 63

Cholelithiasis (P.), 101

"Christian Scientists," 41

Circulatory System (P.), 146, 278, 314

Clinical Society, The (C.), 210

Club Doctor and the Price of Anti-Toxin, The (A.), 273

Cold Tub, The (A.), 77

Collapsible Bath for Use in Pyrexia (A.), 191

Collins and Whitmarsh, The Cases of, 91

Colon, Idiopathic Dilatation of the (P.), 133

Colotomy and Colostomy (P.), 68

Colotomy Lumbar (P.), 133

Commissions (C.), 210

Compulsory Medical Service (C.), 276

Constipation (C.), 260

Consultant. What is a Consultant's Fee? (C.), 228

Consulting Institution, A, 223

Consumption, The Crusade Against (C.), 113

Consumption, Open Air Treatment of (A.), 157

Consumption and Overcrowding (C.), 294

Consumption, Progress of the Crusade Against (A.), 324

Convalescent Homes (W.), 370

Cornea, Treatment of Ulcers of the (C.), 97

Corneal Ulcers, Treatment of (P.), 316

Cottage Homes for the Aged Poor (A.), 375

Cottage Hospital Expenditure (W.), 73

Coxa Vara (C.), 363

Cranial Percussion, The Value of (P.), 115

Craniectomy, Osteoplastic (P.), 116

Criminals, The Children of (P.), 119

Croup, Non-Diphtheritic Membranous (P.), 435

Cystitis (P.), 278

Cystoscopy and Ureteral Cauterisation (P.), 436

Cysts, Pancreatic (C.), 433

Dangerous Illuminants, 358

Darnley Hospital for Infectious Diseases (W.), 218

Death in the Bath (A.), 241

Death, Verification of the Cause of, 59

Decentralisation of Great Cities, The, 140

Dermatology. *See Periscope of Dermatology*

Diabetes (P.), 246

Diagnosis or Proof (C.), 145

Diarrhœa, Infantile (C.), 98

Diet of Prisoners, The, 151

Digestive Organs, Diseases of the (P.), 48, 66, 115

Diphtheria (P.), 162, 178, 196, 212, 220

Diphtheria. Anti-Toxin (A.), 62

Diphtheria Anti Toxin, Late Injections of (C.), 363

Diphtheria Bacilli in Urine (P.), 196

Diphtheria Bacillus, Vitality of the (P.), 196

Diphtheria, Bacteriological Diagnosis of (C.), 47

Diphtheria, Influence of Season and School Attendance on (P.), 196

Diphtheria, Schools for, 156

Diphtheria, Therapy (C.), 212

Disinfection (P.), 367

Dislocations of the Finger, 16

Dispensaries of North-West India (W.), 423

Dispensing by Nurses (C.), 144

Drains and Diphtheria, 205

Dual Consciousness and Allied States (C.), 361

Dysentery (P.), 66

Dyspepsia, The Various Forms of (P.), 48

Ear, Chronic Discharges from (C.), 159, 175

Ectopia Vesicæ (P.), 263

Ectopic Gestation (C.), 363

Eczema (P.), 116, 143

Eczema. What is Eczema? 81

Editor's Letter Box, 15, 39, 89, 105, 122, 137, 169, 187, 254, 306, 337, 355, 372, 425, 442

Education: General and Technical, 323

Electricity, The Dangers of (A.), 125

Employment for the Infirm (A.), 291

Empyema (P.), 417

Endocarditis and Arteritis, Tuberculous (P.), 380

Endocarditis, The Diagnosis of Malignant (C.), 259, 275

Endowment of Research, The, 223

Epilepsy, Bromide of Strontium in (C.), 82

Epilepsy, Surgical Treatment (P.), 163

Erythromelalgia (P.), 346

Ethics and Advertising (A.), 429

Exercise and Over-Exercise, 308

Experimentation and Hospitals, 393, 442

Exploratory Incisions (C.), 378

Extra-Uterine Pregnancy (P.), 179

Facial Paralysis, Congenital (P.), 114

Falkenstein, A Visit to (W.), 54

Feeding of the Children, The (A.), 340

Fibroma, Nasal (P.), 248

Fibro-Myomata of the Uterus (P.), 180

First-Aid Dressing Packets (A.), 4

Fleas as Carriers of Infection (C.), 128

Flies and Infection (C.), 82

Formalin in Blepharitis (P.), 316

Francis Joseph, In Praise of (W.), 219

Friendly Warning, A (A.), 191

Gall Bladder (P.), 365

Gall Bladder, Rupture of (P.), 101

Gall Bladder, Surgery of (P.), 100

Gall-Stones (P.), 332

Galton, Sir Douglas, K.C.B (A.), 410

Gas Stoves in Bed Rooms, 335

Gastric Tetany (P.), 349

Gastric Ulcer (P.), 85

Gastro-Enterostomy (P.), 184, 332

Gastostomy (P.), 332

General Committee and Medical Staff of a Hospital, The Relations of the, 272

General Medical Council, The (C.), 160, 176

Genito-Urinary Surgery (P.), 249, 263, 435

Genu recurvatum (P.), 86

Genu valgum (P.), 87

Glasgow Model Lodging House, 438

Gout and Rheumatism (P.), 346, 400

Government Stamp on Proprietary Medicines, The (C.), 433

Greek in Education (A.), 410

Green Vision in a Tabetic Patient (C), 433

"Grimacing"—"Habit Chorea" (P.), 50

Gynæcology and Obstetrics, Progress in (P.), 164, 179, 297

Habitual Drunkards, 350

Hæmophilia (P.), 130, 401

Hæmorrhage from the Ear (P.), 418

Half-timer, The Anomaly of the (P.), 318

Harveian Oration, The (C.), 64

Heart and Circulation, Diseases of the (P), 380

Heart and its Difficulties, The, 256

Helmholtz, Accommodation Theory of (P.), 316

Heloman (C.), 129

Hepatic Tumours (P.), 100

Home Work and the Factory Acts (A.), 341

Hospital Construction (W.), 36, 218, 253

Hospital Festivals and Meetings (W.), 135, 153, 168, 183, 202, 221, 304, 321, 355, 353, 371, 388, 404, 423, 439

Hospital Housekeeping (W.), 219

Hospital Saturday Fund (W.), 104

Hospital Stamps and Out-patients (W.), 351

Hospital System in Ireland, The (W.), 55

Hospital Trustees, Liability of (W.), 237, 284, 354

Hospitals without House Surgeons (A.), 395

Hydrochloric Acid, Absence of, in Cancer of the Stomach (C.), 415

Icterus, Epileptic (P.), 345

Idioglosia (P.), 331

Idiot Asylums, The Need of (W.), 252

Ill-behaved Patients (A.), 157, 187

Incision, A Preliminary (C.), 312

Inebriates Act, The (A.)

Insane, The Classification of the (W.), 425

Insane, The Story of the, from Year to Year, 57, 121, 253, 269, 304, 422

Insanity, Incipient (A.), 374

Insects and Disease (A.), 4

Institutional Amenities (W.), 72, 88, 105, 120, 137

Intestinal Anastomosis (P.), 132

Intestinal Auto-Intoxication (P.), 115

Intestinal Irrigation, Effects of (P.), 115

Intestinal Obstruction (P.), 132

Intestinal Obstruction, The Treatment of Acute (C.), 112

Intestine, Tumours of the Large (P.), 132

Intestines (P.), 364

Intestines, Surgery of the (P.), 67, 132, 332

Intussusception (P.), 68, 132

Irish Dispensary Doctors (A.), 44

Isolation Hospitals (A.), 273

Jenner, Sir William, 189

Jenner Society, The (A.), 77 *and see p.* 122

Joint Diseases, Chronic (C.), 397, 431

Juvenile Criminals, 79

Kettering General Hospital (W.), 36

Kidney (P.), 249

Kidney, Granular, Some Clinical Aspects of, 343, 377, 413

Kidney, Movable (P.), 278

Kidney Tuberculosis, The Localisation of (C.), 295

Kipling, Mr. Rudyard, 410

Lady Henry Somerset's Home at Duxhurst (A.), 410

Lady Practitioner and Her Sex, The (A.), 125

Laparotomy in Tuberculous Peritonitis (P.), 114

Laryngology (P.), 418, 434

Lead Poisoning, *The Times* on, 42

League of Mercy, The (W.), 386

Legal Restrictions on Practice by Quacks (C.), 161

Leith Public Health Hospital (W.), 303

Lens, Removal of, for Extreme Myopia (C.), 277

Leucocytes, The Nature of (P.), 230

Leukemia (P.), 131

Liability of Hospital Trustees (W.), 237, 284, 354

Liberty and Tuberculosis, 224

Lipoma, Retro-peritoneal (P.), 11

Liquor Trade in America, The, 201, 217, 235, 250

Liver (P.), 332, 364

Liver, Cirrhosis of (P.), 364

Liver, Surgery of the (P.), 100

Locomotor Ataxy (P.), 345

Long Heads and Thick Heads (A.), 109

London Government Bill, The 394

London, The Health of (A.), 257

London Polyclinic, The (A.), 309

London University and the Imperial Institute (A.), 191

London Water Supply, 290

Lunacy in Scotland (W.), 14

Lunatics, The Early Discharge of (A.), 4

Lungs, Diseases of the (P.), 8, 32

Lupus (P.), 83

Magistrate and the Coroner, The (A.), 225

Manchester Children's Hospital Convalescent Home (W.), 370

"Massage" and Immorality (A.), 61

Meanness of the Well-to-Do Man, The (A.), 241

Measles, A Diagnostic Sign of (P.), 9

Measles, Laryngeal Affections in (P.), 435

Medical Advertising (A.), 324

Medical Certificates Again (C.), 228

Medical Certificates, Unreliable, 60

Medical Journalism, New Phases of (A.), 207

Medical Offenders (C.), 415

Medicine in Government, 240

Meningitis, Epidemic Cerebro-Spinal (C.), 327

Metropolitan Provident Medical Association, The (A.), 173

Middle Ear Suppuration (P.), 402, 417

Middlesborough Small pox Epidemic (C.), 211

Midwifery, Aseptic (A.), 6; (P.), 298

Midwifery Forceps, Use and Abuse of (P.), 297

Midwifery and General Practice (C.), 433

Midwifery Practice, The Future of, 124

Midwives Bill, A New, 239

Midwives, The Control of (A.), 46

Midwives, The Employment of, by Medical Men, 108

Midwives, Registration of, 425

Mitral Stenosis, Recurrent Paralysis in (P.), 418

Modern Sociology, 13, 53, 71, 79, 95, 119, 151, 182, 201, 217, 235, 250, 283, 301, 318, 359, 369, 385, 421, 438

Morphia in Cardiac Diseases (P.), 381

Mosquito Theory of Malaria, The (A.), 45

Muddled Consciences (A.), 3

Municipal Amenities (A.), 395

Municipal Slaughter-houses (A.), 45

Mycosis Fungoides (P.), 99

Nasal Secretion, Blue (P.), 248

National Hysteria, 378

National Society for Aid to the Sick and Wounded in War, 39

Nephritis (Interstitial), Prognostic Importance of Eye Lesions in (P.), 317

Nephritis, Malarial (P.), 278

Nerve Changes (P.), 229

Neurology (P.), 314, 330, 345

Neurology, The New (C.), 276

Neurone Theory of the Nervous System (P.), 314

Neuroses Due to Adenoids (P.), 381

Neurosis, Laryngeal (P.), 418

New Appliances and Things Medical, 11, 34, 51, 69, 86, 102, 117, 133, 149, 181, 199, 215, 249, 265, 281, 299, 317, 333, 349, 383, 403, 419, 437

Nitrate of Silver in Conjunctivitis (P.), 316

Nitrous Oxide and Oxygen (C.), 362

Nomenclature, A Study in (C.), 195

"Not at Home" (A.), 359

Notes (W.), 57

Notes and News, 62, 78, 94, 110, 126, 142, 158, 174, 192, 208, 226, 242, 258, 274, 292, 310, 326, 342, 360, 376, 396, 412, 430

Nottingham, The Children's Hospital at (W.), 185, 320, 337, 356, 372

Nursing Streets (A.), 257

Obstetrical Society, The (C.), 65, 210

Occupation as an Element of Diagnosis (A.), 4

Œdema of the Upper Extremity (C.), 127

Œsophageal Pouch (P.), 50

Œsophagus and Stomach, Surgery of the (P.), 50, 84

Old Year, The (C), 243

Oophorectomy for Inoperable Cancer of the Breast (P.), 215

Opening Session, The, 20

Operations on Lunatics (A.), 141

Ophthalmia, Purulent (C.), 293

Ophthalmology, Progress in (P.), 316, 402

Organised Charity Abroad (W.), 236, 252, 268

Orthopædics, Progress in (P.), 33, 68, 85

Otitis Media (P.), 401, 417

Otology (P.), 401

Out-Patient Practice (A.), 340

Ovary, Conservative Surgery of the (P.), 297

Pancreas, Primary Cancer of the (P.), 246

Pancreatic Cysts (C.), 433

Paraldehyde in Asthma (C.), 345

Parasitic Diseases (P.), 99

Partners in Crime (A.), 173

Partnership in General Practice (A.), 5

Pauper, The Competition of the, 385

Pauperism in Whitechapel (A.), 5

Paupers, Incorrigible, Able-bodied (A.), 77

Peculiar People, The (C.), 195

Pelvic Inflammation, The Surgical Treatment of (P.), 164

Pemphigus Neonatorum (P.), 50

Pennyworth of Gas, A, 301

People's Banks, 14, 53, 71

Pericarditis (P.), 381

Periscope of Dermatology (P.), 83, 99, 116, 148

Peritoneal Cavity, Draining the (P.), 10

Peritoneal Tuberculosis (P.), 10

Peritoneum, Subserous Sarcoma of the (P.), 198

Peritoneum, Surgery of the (P.), 10

Peritonitis, Acute Septic (P.), 11

Peritonitis, Tuberculous, Laparotomy in (P.), 114

Pharynx, Diseases of the (P.), 279

Phosphorus Poisoning, 427

Phthisis, Apical, Resection of Ribs in (P.), 145

Phthisis, Alpine Climates for (P.), 32

Phthisis, The Open-air Treatment of (P.), 32; (W.), 54

Phthisis, Prognosis and Diagnosis of (P.), 8

Phthisis, Treatment of Complications of (P.), 32

Physician as a Pessimist, The, 15

"Physician," The Title of, 307

Physique of Schoolboys, The, 139

Plague, The, 75

Plague, Cholera, and other Pestilences (A.), 22

Plague Commission, The (A.), 109

Plague and Quarantine (C.), 96

Plymouth Isolation Hospital, The, 17

Pneumococci (P.), 279

Pneumonia (P.), 416

Pneumothorax (P.), 33

Poisoners, The Stupidity of (A.), 46

Polymyositis (C.), 294

Polyneuritis of Pregnancy and the Puerperium (P.), 346

Post-Diphtheria Paralysis (P.), 196

Post Graduate Teaching (A.), 207

Postage of Periodicals, The (W.), 320

Potts' Disease, Reduction of Deformity in (P.), 33, 85

Potts' Disease, Treatment of (P.), 114

Practical Points (W.), 322

Pregnancy, The Vomiting of (P.), 299

Pregnancy, with Persistent Hymen (C.), 145

Pressure in the Treatment of Wounds (C.), 195

Prince of Wales, The, and a Noble Profession (A.), 341

Prince of Wales's Hospital Fund (W.), 168, 221, 251, 267, 287, 319, 387

Prince's Power for Good, The (W.), 221

Prisoners, The Feeding of (A.), 359

Professional Custom and Professional Caste (C.), 229

Professional Secrecy, 409

Professor on Tour, A (C.), 177

Protargol in Conjunctivitis (P.), 402

Protargol in Ophthalmia Neonatorum (P.), 316

Provident Dispensary, The (C.), 379

Proving too Much (A.), 257

Puerperal Fever, 172

Pulmonary Congestion, Idiopathic (P.), 417

Pulmonary Œdema, Acute (P.), 417

Pulse, The (P.), 380

Purpura and Hæmophilia (P.), 401

Pylorectomy for Cancer (P.), 332

Pyloroplasty (P.), 85

Pylorus, Congenital Hypertrophic Stenosis of the, in Infants, (P.), 114

Pyothorax, Fever following Operations for (P.), 114

Queen's Jubilee Hospital (W.), 335

Quinquennial Census, A Plea for a (A.), 93

Rabbit Bone in Surgery, The Use of (C.), 113

Race against Death, A (A.), 291

Rating of Hospitals (A.), 395

Red Cross in the Soudan, The, 76

Red Cross Society, The (W.), 89

Reform in Criminal Law, 182

Renal Medicine (P.), 262, 278, 296

Respiratory System, Diseases of the (P.), 416

Retro-Peritoneal Fibro-Myoma invading Mesentery (P.), 198

Rheumatism and Gout (P.), 346, 400

Rhinits, Atrophic (P.), 232

Rhinitis, Diphtheritic (P.), 179

Rhinitis, Membranous (P.), 233

Rhinology (P.), 232, 247, 365, 381

Röntgen Rays. *See pp.* 116, 118

Royal College of Surgeons, The (C.), 145

Royal Medical and Chirurgical Society, The (C.), 81, 261

Royal Portsmouth Asylum (W.), 253

St. Bartholomew's Hospital (W.), 121

Salicylic Acid in Granular Conjunctivitis (P.), 403

Sarcoma and Carcinoma, The Treatment of Inoperable (P.), 214

Sarcoma, Interscapulo-thoracic Amputation for (P.), 199

Sarcoma of the Œsophagus (P.), 198

Sarcoma, Subserous of the Peritoneum (P.), 198

Scarlatina, The Pseudo-Membranous Anginas of (P.), 50

Scarlet Fever and Diphtheria (A.), 375

Scarlet Fever, The Etiology of "Return" Cases of, 89

School Board and the Truants, The (A.), 173

Sea-Water for London, 421

Self-Help for Hospitals (W.), 202

Sentimental Sanitation (A.), 141

165462

Serum Exanthemata (P.), 230

Serum Treatment (Diphtheria), (P.), 212

Sewage flooded Cellars (A.), 273

Sherry, The Rehabilitation of (A.), 125

Shock, The Prevention of (O.), 414

Short Leg in Hip Disease (O.), 211

Short Prison Sentences (A.), 95

Sinusitis (P.), 331, 382, 383

Sirdar's Dispatch, The (A.), 22

Slaughter House Question, The (A.), 93

Small-pox, The Control of, by Isolation (A.), 21

Small-pox, The Protection of Convalescents from (A.), 157

Soaps as Disinfectants (P.), 383

Sociology. *See Modern Sociology*

Specifics, The Search for, 92

Specialism, Another: The Consulting Cyclist (A.), 225

Specialists and Practitioners (A.), 61

Spleen, Surgery of the (P.), 100

Splenectomy for Ruptured Spleen (P.), 101

Stammerers (P.), 281

Stamps and Hospitals, 16

State Medicine, Progress in (P.), 264, 367, 382

Stenosis of the Pylorus, Hypertrophic (P.), 114

Stereoscope, The (O.), 194

Sterno-mastoid Tumours (P.), 9

Stomach, Dilatation of the (P.), 349

Stomach, Functions of the (P.), 348

Stomach, Total Extirpation of the (P.), 51

Strumous Glands in the Neck, Treatment of (O.), 65

Student of Medicine, The, 18, 40, 58, 106, 154, 170, 188, 204, 238, 254, 270, 288, 306, 322, 408, 426, 442

Superannuation (A.), 429

"Superheated Dry Air" (O.), 327

Suprarenal Gland Extract (P.), 248

Surgery in Dublin (O.), 227

Surgery, Abdominal (P.), 10, 331

Surgery of the Brain (P.), 101, 115

Surgery, Genito-Urinary (P.), 249, 263, 435

Surgery of the Intestines (P.), 67, 132, 332

Surgery of the Liver, Gall Bladder, and Spleen (P.), 100

Surgery of the Œsophagus and Stomach (P.), 50, 84

Surgery of the Peritoneum (P.), 10

Surgery of the Vermiform Appendix (P.), 147

Swansea Hospital Dispute, The (W.), 173

Sweet Charity's Guide to Christmas Givers (W.), 203

Syphilis (P.), 263

Symphysiotomy (O.), 399

Tetany, Gastric (A.), 5

Therapeutical Notes, 165, 229, 329, 383, 400, 415, 434

Throat, Nose, and Ear, Value of General Treatment in Diseases of the (O.), 345

Thrombosis of Abdominal Vessels (P.), 66

Thymus Hypertrophy and Sudden Death (P.), 9

Tonsils, Chronic Enlargement of (P.), 279

Tonsils, Latent Tuberculosis of the (P.), 279

Tonsilitis (P.), 279

Tramp, The Racial Relations of the, 283

Tropical Diseases, 123

Tuberculosis in Children (O.), 329

Tuberculosis, The Control of, 289

Tuberculosis of the Larynx (P.), 418, 434

Tuberculous Disease of the Intestine (P.), 333

Tumours (P.), 198, 213

Tumours, Vanishing (O.), 398

Typhoid Bacillus and Its Associates, The (O.), 313

Typhoid Fever in Relation to Privies (O.), 98

Typhoid Fever Poison, The Transmission of, Through the Air (A.), 309

Typhoid Fever, The Treatment of (O.), 111

Typhoid Infection (O.), 399

Typhoid Perforation (P.), 67

Typhus Fever (A.), 141

Ulcers, Typhoid, Operations on (O.), 379

Unmarried Women and Maternity Hospitals (A.), 241

Unqualified Assistants, 171

Ureteral Calculi (P.), 249

Urethral Stricture (P.), 263

Ureters, Traumatic Lesions of the (P.), 249

Urinary Disinfectant, A New (O.), 162

Urinary Tract, Septic Infection of the (P.), 435

Urinary Tuberculosis (O), 362

Urination, Vicarious (P.), 249

Urine, On Retention of (O.), 30

Urology (P.), 296

Uterine Displacements (P.), 180

Uterine Myomata, The Treatment of (O), 143 (P.), 180

Uterus, Removal of the, for "Unavoidable Hæmorrhage" (O.), 344

Uterus, The Treatment of Fibro-myomata of the (P.), 180

Uterus, Treatment of Rupture of the (P.), 298

Vaccination, The Government and, 339

Vaccination, Home, 190

Valvular Disease (P.), 278

Venesection in Children's Diseases (P.), 9

Ventilating the Nose (O.), 65

Vermiform Appendix, Surgery of the (P.), 147

Very Old Recipe, A (A.), 109

Vesical Calculus (P.), 436

Victoria Hospital for Children, 254

Virchow, Professor, 19

Vocal Cords, Malignant Disease of the (O.), 82

Voluntary Effort and State Control (A.), 324

Volunteer Medical Department, The, 107

Warning in Regard to the Reduction of Dislocation of the Humerus, A (O.), 434

Water, The Colour of Pure (A.) 44

Water Service, An Object Lesson in Public, 206

Water Supply of East London, The, 1

"White List," A (A.), 324

Whooping Cough, The Paralyses of (P.), 9

Window Accidents (A.), 22

Winter Health Resorts, 23

Women and the Medical Societies (O.), 434

Women as Sanitary Inspectors, 369

Workhouse, The "Attractiveness" of the (A.), 225

Workmen's Compensation Act, (A.), 309, 400

Workmen's Houses Bill (A.), 429

Wounds, The Union of (O.), 194

### THE SPECIAL SECTION FOR NURSES.

Abbey Institute, The, 268

American Letter, Our, 41, 115, 235

Antiseptics for Nurses, 6, xxi, 29, 37, 66, 147, 234, 254

Appointments, 9, xxii, 33, 41, 51, 60, 74, 81, 87, 96, 108, 121, 128, 140, 153, 159, 171, 195, 203, 218, 228, 239, 250, 258, 266

Appointments, Minor, 9, xxvi, 32, 41, 48, 56, 68, 79, 87, 99, 108, 121, 129, 140, 151, 163, 172, 184, 192, 205, 217, 225, 237, 247, 258, 265

Army Nursing Sister, The, 51

Association for Trained Charwomen, 185

Autumn Novelties, xxxiii

Bed Sores, 255

Book and its Story, A, xxxvii, 61, 70, 97, 171, 183, 194, 206, 216, 227, 236, 246, 253, 267

Book World for Women and Nurses, 8, 33, 80, 208, 215, 239

Burns's, Miss, Marriage, 128

Central Poor Law Conference, 225

Chelsea Hospital for Women, 182

Chinese Foot-Binding, 113

Christmas Books, 118, 128

Christmas Entertainments, 139, 149, 158

"Christmas in Hospital," On, 152

Christmas at Malta, A, 115

Christmas Presents, 114

City Orthopædic Hospital, 233

Colonial Nursing Association, The, 106

Cookery Exhibition at the Imperial Institute, 257

County Nursing Association, How to Start a, 264

Death in Our Ranks, 34, 100, 115, 142, 235

Diet for Dyspeptics, 32

District Nursing and Charity, 56

District Nursing of Infectious Diseases, 214

East End Mother's Home, The, 79

Everybody's Opinion, 9, xviii, 43, 62 73, 81, 88, 93, 109 120, 130, 141, 166, 172, 195, 207, 217, 238, 248, 258

Examination Questions for Nurses, 195, 228, 268

"Feed My Lambs," 60

German Letter, Our, 237

Grand Canary as a "Winter Health Resort," 202

Gynæcological Nursing, Notes on, 3, 47, 65

Health Resort in County Clare, A, 186

Help the Nurses to Help the Sick, 120

Hospital Amenities at Norwich, 58

Hospital and Home for Incurable Children, 265

Hospital Secrets, xxxviii

Hospital for Sick Children, Great Ormond Street, 266

How not to Obtain Poor Law Nurses, 226

Humours of Hospital Life, 162

Indian Hospital, In an, 204

Infant Life Protection Act, The, xxxiv

International Conference of Women Workers, 58, 265

Invalid Cookery, 244

Life in a Cottage Hospital, 127

London School of Medicine for Women, xxxiv

London Temperance Hospital, Silver Anniversary of the, 30

Mediæval Practice in the Care of the Sick, 4, xxvi, 42

Medical Relief, Lectures on, 67, 80, 87, 99, 126, 140

Mental Hygiene, 196

"Mental Hygiene as a Basis for Character Formation," 226

Mental Nursing, 88

Milk Laboratories, 245

National Union of Women Workers, 57, 71

New Hospital for Women, The, 247

News from the Nursing World, 1, xv, 27, 35, 45, 53, 63, 75, 83, 93, 101, 111, 123, 136, 145, 155, 167, 177, 189, 199, 211, 221, 231, 241, 251, 261

Norland Nurse, Training and Duties of a, 270

Notes and Queries, 10, 34, 43, 62, 74, 82, 90, 100, 110, 122, 132, 142, 153 164, 176, 186, 198, 210, 220, 230, 240, 250, 260, 270

Novelties for Nurses, 10, lii, 40, 52, 72, 163, 205, 226

Nurses and "Commissions," 79

Nurses' Dances, 109

Nurse's Part in the Prevention of Tuberculosis, The, 214

Nurses for Workhouses, 158

Nursing in Diseases of the Throat, Nose, and Ear, xvii, 55, 77, 85, 93, 103, 125

Nursing in Enteric Fever, 137, 180, 193

Nursing of Sick Children, Hints on the Home, 157, 169, 179, 191, 201, 213, 223, 233, 243, 253, 263

Nursing Hostel, A, 68

Nursing Lectures in a Prison, Some, 224

"Nursing Notes" on Massage, xxvi, 15

Nursing in Paris Hospitals, 5, xxv, 40, 49, 95, 138, 160

Nursing in Switzerland, 105

Opening, An, for Older Nurses: Lecturing, 162

Orphan's Home, Austral Street, S.E., 68

Pasteurising Milk, A Simple Method of, 248

Pension Fund Nurses, The, 140, 152, 159, 169, 183, 195, 205, 227, 260, 265

Pictures from Florence, xli

Pictures from Paris, xxix

Plague at Bombay, 148

Plague Nursing, 7

Poor Law Nursing Question, A, 238

Presentations, 10, 51, 74, 77, 87, 91, 115, 126, 141, 166, 174, 185, 198, 215, 245, 258, 266

Princess Louise at Edinburgh, 106

Problem at Camberwell, The, 163

Puddings for Invalids, 161

Queen Victoria's Jubilee Institute for Nurses, 60, 152

Reading to the Sick. For, 10, xlii, 34, 44, 52, 70, 82, 90, 100, 110, 122, 132, 142, 153, 164, 188, 196, 218, 240, 248, 258

Rio de Janeiro Strangers' Hospital, 96

Royal British Nurses' Association, 50, 117, 173, 203, 239

Royal National Pension Fund for Nurses, 59

St. Alban's Diocesan Nursing Institution, xxxiv

St. John's, Deptford, Opening and Benediction, 73

St John's House, 51

Society for the Protection of Birds, The, 247

Some Superstitions of Southern India, 215

Sutton Cottage Hospital, 201

Temperance Male and Female Nurses' Co-operation, 72

Training in the Provinces, xxii, 31, 39, 48, 69, 78, 86, 94, 104, 129, 159, 170, 181, 192, 203

Travel Notes, 107, 116, 133, 143, 154, 165, 175, 187, 197, 209, 219, 229, 249, 259, 269

Unexpected Cure, An, 218

Useful System of Furnishing, A, 268

Victoria Commemoration Club for Nurses, xxx

Wants and Workers, xli, 39, 52, 60, 65, 78, 87, 110, 128, 141, 195, 219, 228, 247, 260, 266

Warrington Workhouse, 131

West-End Hospital for Diseases of the Nervous System, 235

West London Maternity Home, A, 42

Where to Go, 5, xlii, 43, 61, 68, 78, 87, 94, 106, 115, 128, 151, 176, 226, 247, 267

Women's Industrial Council, 266

Workhouse Infirmaries, The Need of Nursing Reform in, 184

Workhouses or Almshouses? 238

Zenana Bible and Medical Mission, 51

Printed by W. H. and L. Collingridge, City Press, 148 and 149, Aldersgate Street, London, E.C.

**Notices of Births, Deaths, and Marriages** are inserted in this column at a charge of 2s. 6d for an announcement not exceeding four lines.

### NOTICE TO CORRESPONDENTS.

All MS., Letters, Books for Review, and other matters intended for the Editor should be addressed The Editor, "The Hospital," The Hospital Building, 28 & 29, Southampton Street, Strand, W.C. The Editor cannot undertake to return rejected MS., even when accompanied by stamped directed envelopes.

**Notice to Advertisers and Subscribers.**

All Advertisements, Orders for Copies of The Hospital, and Business Communications should be addressed to **THE MANAGER (Not to the Editor),** The Hospital, The Hospital Building, 28 & 29, Southampton Street, Strand, London, W.C.

The Cost of Subscription, post free, is as follows:—Per week, 2½d.; per quarter, 2s. 8d.; per half-year, 5s. 3d.; per annum, 10s. 6d. Foreign:—Per annum, 15s. 2d., payable, in all cases, in advance.

**NOTICE.—Vol. XXIV. of "The Hospital" (April, 1898, to September, 1898), in handsome cloth binding, will shortly be on sale at the Office of this Journal, price 7s. 6d. Cases for binding the Numbers contained in this Volume 1s. 6d. Index to Vol. XXIV., 2½d., post free.**

**The Monthly Part for October is now ready. Price 6d., by post 8d.**

### ANNOTATIONS.

### BOOKS RECEIVED.

Andrew Reid and Co., Newcastle-upon-Tyne.

"Calendar of the University of Durham College of Medicine."

Smith, Elder, and Co.

"First Aid to the Injured." By Dr. Friedrich Esmarch. Translated from the German by H.R.H. Princess Christian.

W. B. Clive.

"Text Book of Zoology." By H. G. Wells, B.Sc.Lond., and A. M. Davies, B.Sc.Lond.

Sampson Low, Marston, and Co.

"Twentieth Century Practice of Modern Medical Science." Edited by Thomas L. Stedman, M.D. Vol. XIV. Infectious Diseases.

"The Treatment of Disease by Electric Currents." By H. S. Morrell, M.D.

F. Bauermeister, Glasgow.

"Methods of Staining the Nervous System." By Dr. Bernhard Pollack. Translated by William R. Jack, M.D., B.Sc.

"Calendar of the School of Medicine of the Royal Colleges, Edinburgh."

Bailliere, Tindall, and Cox.

"A Synopsis of the British Pharmacopœia, 1898." Compiled by W. Wippell Gadd. Third Edition.

"A Pocket Dictionary of Hygiene." By C. T. Kingzett, F.I.C., and D. Homfray, B.Sc.

**Periodicals Received.** — London—Woman's Signal—Dietetic Gazette—Hospital and Sanitarium Record—Trained Nurse—Physical Culture—Westminster Review—Literary Digest—Medical Temperance Review—Asylum News—Medical Record—South African Medical Journal—Guy's Hospital Gazette—Medical Press.

### Plymouth Isolation Hospital.

### Scraps and Gleanings.

The erection of the Leominster Cottage Hospital is to be immediately commenced, a tender of £1,300 having been accepted for the work.

A serious outbreak of typhoid has appeared at Honicknowle, a village near Plymouth. There are 23 cases under treatment. The disease is spreading to adjacent villages.

A dustman recently died of ruptured blood vessels. The medical evidence showed that the lungs were clogged with the dust inhaled in course of the man's employment.

Plans and an estimate for the erection of an isolation hospital for Penarth, at a cost of £4,000, have been forwarded to the County Council, whose decision is now being awaited.

For the first time street collections have been taken at Kettering and Wellingborough in aid of Dr. Barnardo's Homes. £16 odd was collected at Kettering, and £11 odd at Wellingborough.

The first Hospital Sunday collection at Bishop Auckland has been made on behalf of the Lady Eden Hospital, and great interest and much enthusiasm have been shown in the preparations and proceedings.

As a result of the special efforts made by the Hull Hospital Saturday Fund this year the sum of £1,034 has been collected. The Hull Sunday School Union's collections were this year added to those of the Saturday Fund.

It is reported that more than half the boys belonging to the London School Board Industrial School at Brentwood have been suffering from heat stroke, two having succumbed. The school is reported as badly ventilated and over-crowded.

Some twenty-eight or thirty patients from Macclesfield Infirmary were invited by Mrs. Thornycroft, one of the supporters of the infirmary, who extends this invitation each year, to visit her estate one day this summer, where they spent a very enjoyable time.

The trustees of the Yardley Charity Trust have decided to expend a sum of £13,000 at their disposal (which has accrued during the last twenty years) on the purchase of six plots of ground, in all upwards of 30 acres, to be given to the inhabitants of Yardley for use as recreation grounds.

In consequence of objections having been made to the payment by patients of the cost of disinfecting bedding, &c., at the Woodbridge Hospital, Guildford, it has been decided, on the recommendation of the General Purposes Committee, that no charge shall be made by the board in respect of the cost of such disinfection.

The Borchardt Ward of the Manchester Children's Hospital, which has hitherto been used by the Manchester Corporation for fever cases, and has now been handed back to the Board of Management for general cases, was formally opened on September 12th. The new ward has 28 beds, and has cost about £800 for alterations.

The *New Penny Magazine* is the title of Messrs. Cassell and Company's new weekly periodical, of which the first number is to appear on October 19th. It will provide a greater amount of reading matter and illustrations than has been hitherto given for a penny in any magazine. Each issue is to contain 64 large pages, fully illustrated.

It is hoped by the Committee of the Bristol General Hospital that they may be enabled to improve the kitchen department of their institution. It is estimated that this improvement, which is urgently needed, would cost about £1,000. Other improvements are also in contemplation at this institution if the necessary donations can be obtained.

The chairman of a rural district council, being evidently of a peace-loving disposition, has intimated that all the members are quite at liberty to indulge in the weed during the transaction of the business of the council, and he considers that if this rule prevailed at meetings of public bodies, they would have less wrangling and fewer exhibitions of temper.

Leo, the beautiful dog who was exhibited in London last June, and was visited by the Princess of Wales, has been pressed into the service of the County and City of Cork Hospital for Women and Children, and at the Ballsbridge Horse Show Leo might have been seen collecting money towards the reduction of the debt of £1,000 incurred by this hospital for the sanitary improvements which have been introduced.

Does the payment of £50 to an architect for drawing up plans, specifications, &c., for a cottage hospital preclude his being styled "honorary architect" in connexion with that particular piece of work? Some of the committee of the Leominster Cottage Hospital are very positively of opinion that it does, but as notice had not been given of their motion to alter the term "honorary architect" in the minutes to "architect" simply, it was allowed to drop.

Paris has been recently somewhat alarmed by the discovery of the business methods of a certain chemist in a populous quarter of Paris, whose custom it is stated to have been to substitute for any costly drugs contained in a prescription others of cheaper kind, and to omit altogether the expensive ones. By this means he was enabled to considerably undersell other chemists. The police are now on his track, and it is stated that inquiries seem to indicate that such frauds are comparatively common.

It is stated that Thomas Guy, the beneficent founder of Guy's Hospital, was in private life very parsimonious. He even rebelled at the thought of lighting a farthing candle, preferring to sit in the dark, as he did on one occasion when a friend, also of parsimonious habits, came to ask him for a lesson on frugality; whereupon Thomas Guy, on learning the object of his friend's visit, promptly extinguished the farthing light, and then proceeded to give his advice in the dark. Example is better than precept, was evidently Guy's motto.

The Committee of the Cork Hospital Saturday Fund have been suffering from the excessive importunity of their collectors, which has disgusted the inhabitants of Cork to such an extent that the street collections have been gradually declining, and it was suggested that on this account they should this year be omitted, unless, the Committee stated, the various institutions benefiting would take more interest in the collection. It has been decided, therefore, to have the 1898 collection as usual, steps being taken to reorganize the arrangements and make things more satisfactory all round.

### The Student of Medicine.

### APPOINTMENTS.

C. H. Bond, D.Sc., M.D., Ch.M.Edin., appointed Senior Assistant Medical Officer to the new London County Asylum, Bexley, Kent. G. Cowie, M.B.Aberd., appointed Medical Officer to the Holborn Union Workhouse, City Road. J. Dallewy, M.R.C.S.Eng., L.R.C.P.Lond., appointed Medical Officer of Health for the Wem Rural District Council. W. A. Densham, M.R.C.S., L.R.C.P., appointed House Surgeon to Charing Cross Hospital. C. F. Downman, L.R.C.P.Lond., M.R.C.S., appointed Medical Officer of Health to the Burnham-on-Crouch Urban District. D. P. Fitzgerald, B.A., M.B., R.U.I., appointed Demonstrator of Anatomy in the Queen's College, Cork. D. McD. Forbes, F.R.C.S.Edin., L.R.C.P., appointed Medical Officer of Health to the Eastwood Urban District. A. H. Gibbon, L.R.C.P., L.R.C.S.Edin., L.R.P.S.Glasg., appointed Medical Officer for the St. Mary's Parish, and Public Vaccinator for the Borough of Bury. G. C. Hayes, F.R.C.S.Eng., appointed House Surgeon to the Royal Westminster Ophthalmic Hospital. A. S. Hedley, M.B. and B.S Durh., appointed Medical Officer to the Rothbury Workhouse, and Medical Officer and Public Vaccinator of the Eastern District of the Rothbury Union. G. G. Morrice, M.A., M.D.Cantab., M.R.C.P., appointed Consulting Physician to the Salisbury Infirmary. H. J. Paterson, M.A., M.B.Cantab., F.R.C.S.Eng., appointed Assistant Medical Officer to the Hospital of St. Francis, 145, New Kent Road, S.E. H. Peck, M.B. and C.M.Edin., D.P.H.Camb., appointed Medical Officer of Health to Chesterfield Rural District Council. J. S. Revely, M.D., M.R.C.S., appointed Medical Officer to the Tettenhall District of the Seisdon Union. W. St. A. St. John, M.R.C.S.Eng., L.R.C.P.Lond., appointed Assistant House Surgeon to the Derbyshire Royal Infirmary. Dr. W. H. Smith, appointed Medical Officer for the Fourth District of the Brackley Union. J. R. Black, M.D.Edin., appointed Visiting Surgeon to the Greenock Infirmary. J. F. Brown, M.D., C.M., appointed Medical Officer of the Fourth District of the Thingoe Union. J. Chestnutt, B.A., L.R.C.S., L.R.C.P.Edin., appointed Medical Officer to Howden Union Workhouse and District, East Yorkshire. J. W. Eastment, M.R.C.S.Eng., L.R.C.P., appointed Medical Officer for the Fourth District of the Saffron Walden Union. R. K. Ellis, M.B., B.Ch., appointed Medical Officer for the Third District of the Southwell Union. F. S. Eve, F.R.C.S., appointed Surgeon to the London Hospital. G. C. Henderson, M.R.C.S., L.S.A., appointed Chief Medical Officer for Zululand, and District Surgeon for Eshowe Division, Zululand. Dr. M. T. Kelly, appointed Medical Officer of the Workhouse and the Coddenham District of the Bosmere and Claydon Union. J. W. Leitch, M.A., M.B., Ch.B.Glasg., appointed Junior Assistant Medical Officer to the Counties Asylum, Carlisle. J. McCarthy, F.R.C.S., appointed Consulting Surgeon to the London Hospital. Ethilda Meakin, M.B., appointed Junior Medical Officer to the Camberwell Infirmary. D. K. Muir, L.R.C.P., L.R.C.S.Edin., appointed Medical Officer for the South Longbenton District of the Tynemouth Union. W. H. Pimblett, M.B. and C.M.Edin., appointed Medical Officer for the Seventh District of the Preston Union. A. C. Renwick, M.B., Ch.B.Edin., appointed House Surgeon to Noble's Isle of Man Hospital, Douglas. Dr. Richards, appointed Medical Officer of Health for Dronfield. D. S. Sleith, B.A., M.B., B.Ch., B.A.O. T.C.D., L.M., appointed Assistant Resident Medical Officer in the North Dublin Union. W. C. Sprague, M.D.Edin., M.R.C.S.Eng., appointed Medical Officer for the Seventh District of the Saffron Walden Union. E. D. Warren, M.R.C.S., L.R.C.P., appointed Senior Assistant Medical Officer to the Counties Asylum, Carlisle. E. B. Wrench, M.B., B.C.Cantab., appointed Medical Officer for the Baslow District of the Bakewell Union.

The following is the list of medallists at St. Thomas's Hospital Medical School: Practical medicine, E. F. Buzzard; surgery and surgical anatomy, S. O. Bingham; pathology and morbid anatomy, A. W. Sikes; Grainger testimonial prize, R. Beer; for reports of surgical cases, O. W. Pilcher; for reports of cases in obstetric medicine, A. Bevan; for general proficiency and good conduct, H. E. Hewitt.

28 & 29, SOUTHAMPTON STREET, STRAND, LONDON, W.C.

